# Rapid Molecular Diagnostics for MDR Nosocomial Infections in ICUs: Integration with Prevention, Stewardship, and Novel Therapies

**DOI:** 10.3390/diagnostics15233060

**Published:** 2025-11-30

**Authors:** Karina Cristina Marin, Stelian Adrian Ritiu, Adelina Băloi, Claudiu Rafael Barsac, Dorel Sandesc, Marius Papurica, Alexandru Florin Rogobete, Daiana Toma, Mirela Tamara Porosnicu, Ciprian Gindac, Madalina Butaș, Ovidiu Horea Bedreag

**Affiliations:** 1 Department of Otorhinolaryngology, Victor Babeş University of Medicine and Pharmacy, 300041 Timişoara, Romania; marin.karina@umft.ro; 2 Faculty of Medicine, Victor Babeș University of Medicine and Pharmacy, 300041 Timișoara, Romania; stelian.ritiu@umft.ro (S.A.R.); bedreag.ovidiu@umft.ro (O.H.B.); 3 Clinic of Anaesthesia and Intensive Care, Emergency County Hospital “Pius Brînzeu”, 300723 Timișoara, Romania; 4 Doctoral School, Victor Babe University of Medicine and Pharmacy Timisoara, Eftimie Murgu Square 2, 300041 Timisoara, Romania; 5 Anaesthesia and Intensive Care Research Center (CCATITM), Victor Babeș University of Medicine and Pharmacy, 300041 Timișoara, Romania

**Keywords:** multidrug-resistant pathogens, nosocomial infections, antimicrobial stewardship, rapid molecular diagnostics, UNYVERO, GeneXpert, novel antimicrobials, carbapenem-resistant Acinetobacter

## Abstract

**Background/Objectives**: Multidrug-resistant (MDR) nosocomial infections remain a major challenge in intensive care units (ICUs), where delays in diagnosis and suboptimal antimicrobial therapy significantly impact outcomes. This narrative review synthesizes international literature and local epidemiological data from Western Romania to examine the role of rapid molecular diagnostics in the management of MDR infections and their integration with prevention and antimicrobial stewardship (AMS) strategies. **Methods**: Evidence was collected through a narrative literature review using PubMed, WHO, and ECDC sources published between 2010 and 2025. Key terms included “rapid molecular diagnostics,” “sepsis,” “ICU,” “UNYVERO,” “GeneXpert,” “BioFire,” and “carbapenem resistance.” Studies were selected based on clinical relevance to rapid diagnostics and MDR pathogens; no PRISMA-based systematic methodology was applied. **Results**: Diagnostic performance varies by platform and clinical syndrome. UNYVERO Hospitalized Pneumonia panel demonstrates a sensitivity range of 88.8–91.4% and specificity of 94.9–99.5% in respiratory infections, with a turnaround time of approximately 4–5 h. The GeneXpert Carba-R assay identifies major carbapenemases within 45–60 min with reported sensitivity 96–100% and specificity of 93–99%. BioFire^®^ Pneumonia and Blood Culture Identification panels similarly provide rapid syndromic results within 1 h, enabling earlier optimization of antimicrobial therapy. Local ICU data from Western Romania identified a substantial burden of carbapenem-resistant *Acinetobacter baumannii*, underscoring the need for rapid resistance detection to guide therapy. **Conclusions**: Rapid molecular diagnostics, when integrated with prevention bundles and AMS programs, facilitate earlier targeted therapy, support responsible antimicrobial use, and improve clinical decision-making in MDR infections. Their value is amplified in settings with high resistance prevalence. Wider implementation, combined with surveillance and access to novel antimicrobials, is essential to improve outcomes in critically ill patients.

## 1. Introduction

Antimicrobial resistance (AMR) has become one of the most urgent global health threats, with multidrug-resistant (MDR) organisms responsible for an estimated 4.95 million associated deaths in 2019 and projected to cause up to 10 million deaths annually by 2050 if no decisive interventions are implemented. Intensive care units (ICUs) represent the epicenter of this crisis, accounting for disproportionately high rates of nosocomial infections due to the widespread use of invasive procedures, broad-spectrum antimicrobials, and the vulnerability of critically ill patients. Globally, MDR Gram-negative bacteria such as *Acinetobacter baumannii*, *Pseudomonas aeruginosa*, and carbapenem-resistant Enterobacterales (CRE) have been identified as priority pathogens by the World Health Organization, given their escalating prevalence and limited therapeutic options [1].

Despite these concerns, conventional diagnostic methods rely heavily on culture-based identification and susceptibility testing, requiring 48–72 h to deliver actionable results. This diagnostic delay contributes to inappropriate empiric therapy, prolonged ICU stay, higher mortality, and exacerbation of antimicrobial resistance. Over the last decade, rapid molecular diagnostics—including PCR-based syndromic panels—have shown the potential to reduce this gap by enabling early pathogen identification and resistance gene detection within hours, thereby supporting timely targeted therapy and improved antimicrobial stewardship (AMS) [2].

However, several literature gaps remain. First, most studies focus on the analytical performance of syndromic PCR platforms but provide limited evidence regarding their real-world clinical impact on therapeutic decisions, ICU mortality, and antibiotic consumption, especially in low- and middle-income countries [3]. Second, data on the integration of rapid molecular diagnostics with prevention bundles, AMS interventions, and access to novel antimicrobials remain scarce, although these components must function synergistically to reduce the burden of MDR infections. Third, geographical variability in MDR epidemiology is significant, yet few studies incorporate local microbiological data into broader analyses of diagnostic strategies. Eastern Europe—particularly Romania—remains underrepresented in the global AMR literature despite some of the highest rates of carbapenem resistance in the EU [4].

To address these gaps, this narrative review integrates global evidence with local epidemiological data from Western Romania, focusing on the role of rapid molecular diagnostics within a broader strategy encompassing infection prevention, antimicrobial stewardship, and novel therapeutic options for MDR nosocomial infections in ICUs.

## 2. Antimicrobial Stewardship

Antimicrobial stewardship has evolved over the past decades as a cornerstone of modern healthcare policy and clinical practice. Far from being a restrictive measure aimed at reducing antibiotic availability, stewardship embodies a multidimensional framework designed to ensure rational, evidence-based use of antimicrobials. Its ultimate goal is to optimize clinical outcomes while minimizing unintended consequences such as toxicity, disruption of the commensal microbiota, and the selection and dissemination of resistant organisms.

The fundamental principles of AMS emphasize prescribing antibiotics only when clearly indicated, selecting the most appropriate agent, and administering it at the correct dose, via the optimal route of administration, for the recommended duration. This rational approach not only benefits individual patients by preventing unnecessary drug exposure but also safeguards the collective effectiveness of antibiotics as a finite global resource [5,6].

Modern stewardship programs employ a variety of strategies:

Empirical therapy with rapid de-escalation: initiating broad-spectrum coverage in life-threatening infections followed by narrowing or discontinuation once microbiological data become available.

Antibiotic cycling or rotation: periodically alternating classes of antibiotics within a clinical unit to reduce selective pressure for specific resistance mechanisms.

Formulary restriction and pre-authorization: limiting the use of certain last-line antimicrobials (e.g., carbapenems, novel β-lactam/β-lactamase inhibitor combinations) to cases with microbiological or clinical justification.

Audit and feedback systems: regular review of prescriptions with direct feedback to clinicians, fostering a culture of accountability and continuous improvement.

At the hospital level, AMS programs are inseparably linked with infection prevention and control (IPC). Surveillance and regulation of antibiotic use across the institution must be coupled with strict hygiene measures and device-associated infection prevention protocols. This integration is particularly critical in intensive care units and emergency departments, where invasive devices such as central venous catheters, endotracheal tubes, and urinary catheters are frequently used. These devices represent major risk factors for catheter-related bloodstream infections, ventilator-associated pneumonia (VAP), and catheter-associated urinary tract infections (CAUTIs). Standardized care bundles for insertion, maintenance, and timely removal have been shown to substantially reduce infection incidence and consequently decrease antimicrobial consumption. Evidence from multicenter studies confirms the impact of stewardship interventions. For example, implementation of AMS programs in European ICUs has been associated with significant reductions in broad-spectrum antibiotic use, shorter treatment durations, lower rates of *Clostridioides difficile* infection, and a measurable decline in MDR organism prevalence. Globally, AMS has been endorsed by the WHO as a central pillar in combating antimicrobial resistance, emphasizing the need for integration of stewardship principles into national health systems (Table 1).

**Table 1:** ICU diagnostic–therapeutic playbook integrating empiric therapy, rapid testing, and stewardship rules.

Step	Action	Details/Examples
A. Empiric Start (Syndrome-based)	HAP/VAP	Meropenem ± vancomycin (depending on MRSA prevalence)
	Severe sepsis/BSI	Meropenem ± amikacin; consider ceftazidime–avibactam in high-KPC units
	IAI (severe)	Piperacillin-tazobactam or meropenem; adjust for ESBL prevalence
B. Rapid Test Triage	Choose cartridge	UNYVERO HPN (respiratory; TAT 4–5 h) UNYVERO BCU (blood culture; TAT 4–5 h) GeneXpert Carba-R (MBL/KPC/OXA detection; TAT 45–60 min).
C. Gene-Based Therapeutic Rules	KPC detected	Start ceftazidime–avibactam or meropenem–vaborbactam. Avoid carbapenem monotherapy
	NDM detected	First-line: aztreonam–avibactam alternative: cefiderocol.
	OXA-48-like detected	Start ceftazidime–avibactam; alternative cefiderocol.
	*mec*A/*me*cC detected	Switch to vancomycin or linezolid
D. When MIC Confirmation is Needed	ESBL/AmpC	Confirm MIC before using cefepime or piperacillin–tazobactam
	OXA-48-like	Requires phenotype confirmation due to variable hydrolysis
	Any escalation to new agents	Ceftolozane–tazobactam, cefiderocol, and imipenem-relebactam require MIC confirmation before use

Taken together, antimicrobial stewardship is not merely a clinical guideline but a comprehensive, system-wide strategy. Its success depends on the coordinated efforts of clinicians, microbiologists, pharmacists, infection control specialists, and policymakers. By aligning rational prescribing practices with robust preventive measures, AMS represents one of the most powerful tools available to curb the tide of antimicrobial resistance and preserve therapeutic options for future generations [7,8,9,10].

## 3. Prevention Strategies for Nosocomial Infections

### 3.1. Prevention of Central Line–Associated Bloodstream Infections (CLABSIs)

Central venous catheters (CVCs) are indispensable in the management of critically ill patients, but their use is associated with a significant risk of central line–associated bloodstream infections (CLABSIs). Evidence-based prevention strategies, integrated into standardized insertion and maintenance bundles, have consistently demonstrated reductions in infection rates. The following measures are recommended:

Risk–benefit assessment: The decision to place a CVC should consider both infection risk and mechanical complications. Femoral access carries the highest infection risk, whereas the subclavian site has the lowest, though it is associated with higher rates of mechanical complications such as pneumothorax and hematoma.

Site selection: Whenever feasible, the subclavian vein should be prioritized to minimize infectious risk, while avoiding unnecessary femoral insertions.

Ultrasound guidance: The use of echography-guided techniques is strongly recommended to reduce the number of puncture attempts and procedural complications.

Catheter choice: Select the catheter with the minimum number of lumens required for patient management, thereby limiting infection risk.

Replacement after emergency insertion: Catheters placed under suboptimal aseptic conditions (e.g., in emergency settings) should be replaced as soon as possible, ideally within 48 h.

Hand hygiene and aseptic technique: Rigorous handwashing or hand disinfection prior to donning sterile equipment is mandatory. Catheter insertion must always follow a maximal aseptic technique.

Skin antisepsis: Chlorhexidine 2% in an alcohol-based solution is superior to other antiseptics and should be used for skin preparation.

Use of full barrier precautions: Insertion should be performed under full sterile barrier conditions, including sterile gown, gloves, mask, cap, and a large sterile drape covering the patient from head to toe. Any assisting personnel should also wear masks and caps.

Daily evaluation of necessity: The continued need for the CVC must be reassessed daily, and catheters should be removed as soon as they are no longer essential.

Port disinfection: All catheter hubs and injection ports should be disinfected with alcohol or chlorhexidine prior to each use.

Site monitoring: The insertion site should be inspected daily for signs of local infection, including erythema, tenderness, edema, purulent discharge, and dressing integrity.

Accessory replacement: Replace CVC accessories (tubing, stopcocks, etc.) at recommended intervals—every 6–12 h for propofol infusion, within 24 h for parenteral nutrition and blood products, and at least every 96 h for crystalloids.

Dressing management: Transparent semipermeable dressings should be used to cover the insertion site. Dressings must be changed at least every 7 days, or earlier if compromised (wet, soiled, or loose).

Avoid routine replacement: Routine scheduled replacement of CVCs (e.g., every 7 days) is not recommended, as it increases the risk of introducing infection. When replacement is necessary, guidewire-assisted exchanges should be avoided to prevent contamination of the new catheter.

Implementation of these preventive strategies, particularly when applied as a bundled intervention, has been shown in multicenter trials to significantly reduce the incidence of CLABSIs, improve patient outcomes, and decrease healthcare costs [11,12,13].

### 3.2. Prevention of Ventilator-Associated Pneumonia (VAP)

Ventilator-associated pneumonia (VAP) remains one of the most frequent and severe hospital-acquired infections in intensive care units, contributing significantly to morbidity, mortality, and healthcare costs. In order to reduce its incidence, preventive strategies endorsed by the Romanian Society of Anesthesia and Intensive Care (SRATI), aligned with the International Society for Infectious Diseases (ISID) 2024 updated recommendations, should be systematically implemented [14].

The key evidence-based measures include the following:

Avoidance of intubation and reintubation whenever possible: The use of high-flow nasal oxygen therapy or non-invasive ventilation is recommended as an alternative to invasive mechanical ventilation in eligible patients, as these strategies have been associated with reduced rates of VAP.

Head-of-bed elevation: Patients on mechanical ventilation should be maintained in a semi-recumbent position (30–45°). This reduces the risk of aspiration of gastric contents and lowers the incidence of VAP compared with supine positioning.

Oral hygiene: Regular oral care every six hours is strongly advised. Current evidence suggests that mechanical cleaning is more effective and safer than chlorhexidine-based oral decontamination, which has been associated with potential adverse effects in critically ill patients.

Early enteral nutrition: Enteral feeding should be initiated as early as possible, as it preserves gut integrity and reduces the risk of bacterial translocation compared with parenteral nutrition.

Ventilator circuit management: Ventilator circuits should not be routinely changed. They should only be replaced if visibly soiled, malfunctioning, or at the manufacturer’s recommended intervals, as unnecessary changes increase the risk of contamination.

Use of antimicrobial filters: Where available, antimicrobial filters may be employed in ventilator circuits to reduce microbial colonization and subsequent infection risk.

Closed-system suctioning: Airway suctioning should be performed under strict aseptic conditions, preferentially using closed suction systems. These reduce the risk of environmental contamination and maintain positive end-expiratory pressure (PEEP), thereby lowering the risk of hypoxemia and infection.

Collectively, these interventions form part of the VAP prevention bundle. Multiple multicenter studies have demonstrated that strict adherence to such bundles results in a substantial reduction in VAP incidence, shortened duration of mechanical ventilation, reduced ICU length of stay, and improved patient survival [15,16].

Additional Interventions with Limited Strength of Evidence

Beyond the core VAP prevention bundle, several adjunctive interventions have been proposed. While these measures may be beneficial in selected clinical settings, they generally lack high-grade evidence from large randomized controlled trials and are therefore recommended with caution [17,18]:

Selective oral or digestive decontamination: In healthcare systems or regions with a low prevalence of multidrug-resistant (MDR) organisms, selective decontamination of the oropharynx or gastrointestinal tract using non-absorbable antimicrobials has been associated with reduced rates of VAP. However, its use remains controversial due to concerns about promoting resistance in high-MDR environments.

Endotracheal tubes with subglottic secretion drainage: For patients expected to require prolonged mechanical ventilation (>72 h), the use of endotracheal tubes equipped with a subglottic suction port may reduce microaspiration of secretions, thereby lowering the incidence of early-onset VAP.

Early tracheostomy: In patients anticipated to require prolonged mechanical ventilation, early conversion from endotracheal intubation to tracheostomy has been suggested to decrease airway trauma, facilitate weaning, and potentially reduce the risk of VAP. Evidence remains mixed, with variability in practice across institutions.

Post-pyloric feeding: In patients at high risk of aspiration (e.g., those with impaired gastric emptying or severe neurological conditions), placement of a post-pyloric feeding tube rather than gastric feeding may reduce aspiration events and consequently lower the risk of pneumonia.

While these interventions are supported by observational studies and some randomized trials, their overall quality of evidence is low to moderate. As such, they should be implemented selectively, tailored to patient characteristics, institutional epidemiology, and resource availability [19,20].

Emerging and Investigational Measures for VAP Prevention

In addition to established and adjunctive interventions, several preventive strategies for ventilator-associated pneumonia (VAP) are currently under investigation. Although these measures have not yet demonstrated consistent reductions in VAP incidence in large-scale clinical trials, they remain of interest in ongoing research and may provide future benefit [21].

Endotracheal tubes with ultrathin cuffs: Designed to reduce microaspiration around the cuff, these devices may theoretically limit pathogen entry into the lower respiratory tract. However, evidence supporting their efficacy remains inconclusive.

Endotracheal tubes with tapered (conical-shaped) cuffs: Similarly, tapered cuffs have been proposed to improve sealing of the tracheal lumen and reduce aspiration risk. Current data are heterogeneous and insufficient to confirm a reduction in VAP incidence.

Chlorhexidine body washing: Daily whole-body bathing with chlorhexidine has been hypothesized to decrease colonization with resistant organisms and secondary infection. While effective in reducing bloodstream infections in some studies, its impact on VAP specifically remains uncertain.

Monitoring of gastric residual volume (GRV): Routine GRV measurement has been evaluated as a strategy for reducing aspiration risk in mechanically ventilated patients receiving enteral nutrition. Recent trials suggest that systematic GRV monitoring may not significantly influence VAP incidence and may unnecessarily interrupt feeding.

Early initiation of parenteral nutrition: Although intended to prevent aspiration associated with enteral feeding, early parenteral nutrition has not been shown to reduce VAP rates and may increase the risk of bloodstream infection and metabolic complications.

Automatic cuff pressure monitoring: Continuous electronic monitoring of endotracheal tube cuff pressure may provide more stable control than manual checks, theoretically reducing microaspiration. However, current evidence is limited to small-scale studies.

Frequent manual cuff pressure monitoring: While regular measurement is part of standard practice to avoid both under- and over-inflation, the optimal frequency and its direct effect on VAP prevention remain under evaluation.

Taken together, these strategies highlight the evolving nature of VAP prevention research. While they are biologically plausible and technically feasible, robust evidence is lacking, and none are currently recommended as routine measures. Future randomized controlled trials will be required to clarify their role in comprehensive prevention bundles [22,23].

### 3.3. Prevention of Catheter-Associated Urinary Tract Infections (CAUTIs)

Catheter-associated urinary tract infections (CAUTIs) represent one of the most frequent healthcare-associated infections worldwide, contributing significantly to patient morbidity, prolonged hospital stays, and increased healthcare costs. Preventive strategies, supported by international guidelines such as those from the Centers for Disease Control and Prevention (CDC) and the Infectious Diseases Society of America (IDSA), are essential in minimizing their incidence [24].

Key recommended measures include the following:

Avoidance of unnecessary catheterization: Urinary catheters should only be placed when strictly indicated. Alternative strategies such as intermittent catheterization or external collection devices should be considered whenever possible.

Strict aseptic insertion technique: Catheter placement must always adhere to rigorous aseptic protocols, including hand hygiene, sterile equipment, and sterile barrier precautions.

Proper catheter and drainage system positioning: The urinary catheter should be secured above the thigh, and the drainage bag must always be positioned below the level of the bladder to prevent retrograde flow of contaminated urine.

Prevention of environmental contamination: The drainage bag must never come into contact with the floor to avoid bacterial colonization.

Catheter replacement: Foley catheters should be changed at regular intervals (no longer than 14 days), and immediately in the event of infection, obstruction, or breach of system integrity (e.g., disconnection between catheter and drainage bag).

Prompt removal of the catheter: The indwelling catheter should be removed as soon as it is no longer essential, as duration of catheterization is the single most important risk factor for CAUTIs.

Implementation of these measures, particularly as part of structured CAUTI prevention bundles, has been shown to significantly reduce infection rates in both acute and long-term care facilities. Daily evaluation of catheter necessity and strong institutional policies on device management remain the cornerstones of prevention [25,26].

### 3.4. General Measures to Prevent Horizontal Transmission of Bacterial Pathogens

In high-density hospital environments such as intensive care units (ICUs) and emergency departments, where patient isolation is not always feasible, horizontal transmission of multidrug-resistant organisms (MDROs) represents a major threat. General preventive measures, designed to interrupt cross-transmission, are essential to maintain patient safety and reduce nosocomial infection rates [27].

The most important strategies include the following:

Restrictive and rational antibiotic use: Antimicrobials should only be prescribed when clinically indicated, in line with antimicrobial stewardship principles. This reduces selective pressure and helps prevent the emergence and dissemination of resistant strains [7,8,9].

Environmental cleaning and disinfection: Daily cleaning and disinfection of patient care areas, with particular attention to high-touch surfaces, is mandatory to minimize microbial reservoirs in shared clinical spaces [28,29].

Appropriate use of personal protective equipment (PPE): Healthcare personnel must consistently wear gowns, masks, caps, and non-sterile gloves when caring for patients. Correct donning and doffing procedures are critical to reduce self-contamination [30,31].

Hand hygiene: Rigorous handwashing or alcohol-based hand disinfection must be performed in accordance with World Health Organization (WHO) recommendations, particularly before and after contact with patients or their environment [27,32].

Glove use: Non-sterile or sterile gloves should be used depending on the type of patient contact. Sterile gloves must be worn after proper hand hygiene when contact with mucous membranes, non-intact skin, or aseptic procedures is anticipated [33,34].

Protective practices during patient hygiene care: Staff should wear hydrophobic gowns during bathing procedures and change gloves when moving from contaminated to clean areas of the patient’s body, in order to avoid cross-contamination [33,34].

Strict cleaning and disinfection of medical spaces: Regular monitoring and enforcement of environmental cleaning protocols in ICUs and emergency departments are essential components of infection control [28,29,35].

Collectively, these measures represent the foundation of infection prevention and control in non-isolated, high-risk clinical areas. Their consistent implementation has been shown to reduce the horizontal spread of MDROs and improve overall patient outcomes [34,36].

## 4. Early Detection of Pathogens and Timely Empiric Therapy

Awareness of the most common pathogens implicated in community-acquired and hospital-acquired infections is essential to ensure the prompt initiation of appropriate therapy in septic patients. Numerous studies have consistently demonstrated that mortality in severe, life-threatening infections increases in direct proportion to the delay in administering an effective antimicrobial agent [37]. Early and accurate empiric therapy therefore represents a cornerstone of sepsis management and is strongly associated with improved survival.

To guide the initial choice of antibiotics, clinicians rely on a combination of international evidence-based guidelines and local epidemiological data. Empiric therapy must be tailored not only to the suspected source of infection but also to local resistance patterns, ensuring adequate coverage of the most likely pathogens. Once microbiological results and susceptibility data become available, therapy should be de-escalated to narrower-spectrum agents, thereby balancing effective treatment with antimicrobial stewardship principles.

This dynamic approach—rapid empiric initiation followed by rational de-escalation—remains the standard of care in managing septic patients. It enables clinicians to provide timely, life-saving therapy while simultaneously mitigating the selective pressures that drive antimicrobial resistance [38].

In the following section, we discuss the recommended approach when managing patients with infections suspected to be caused by multidrug-resistant (MDR) organisms. The prolonged turnaround time of conventional microbiological cultures and susceptibility testing—typically a minimum of 48–72 h—necessitates that initial antimicrobial therapy in emergency and critical care settings be instituted empirically. This empiric treatment is guided by knowledge of the pathogens most frequently implicated in specific clinical syndromes, their local susceptibility profiles, and, where available, the results of rapid diagnostic tests.

Prior to the availability of culture and susceptibility data, several rapid diagnostic tools can assist in guiding therapy. The simplest and most cost-effective method remains Gram staining, which provides valuable information on bacterial morphology (cocci, diplococci, bacilli) and cell wall characteristics (Gram-positive vs. Gram-negative). For example, Gram-positive cocci in clusters are suggestive of *Staphylococcus aureus*, encapsulated Gram-positive diplococci may indicate *Streptococcus pneumoniae*, small Gram-negative cocci may correspond to *Neisseria* species, and coccobacilli may suggest *Haemophilus influenzae*.

However, Gram staining has important limitations. While it allows for a preliminary morphological classification, it cannot distinguish between aerobic and anaerobic organisms, nor does it provide any information on antimicrobial susceptibility. Resistance patterns within the same bacterial species may vary significantly, primarily due to the acquisition of resistance mechanisms. These mechanisms are frequently driven by repeated or inappropriate exposure to antimicrobials, such as suboptimal dosing or inadequate duration of therapy [39].

Consequently, while Gram staining may aid in the early identification of likely pathogens, definitive therapeutic decisions must await confirmatory microbiological cultures and antibiograms, or be supplemented by more advanced rapid molecular diagnostic methods [40].

Although Gram staining remains a simple and rapid method for preliminary classification of microorganisms, additional microbiological tools now play a critical role in the early diagnostic process. Direct microscopic examination, including Ziehl–Neelsen staining for mycobacteria or India ink for fungal pathogens, can provide immediate clues in selected clinical scenarios. Conventional culture techniques continue to be essential for definitive identification and susceptibility testing; however, they are limited by the prolonged turnaround time of 48–72 h, which often necessitates empiric broad-spectrum therapy at presentation [41].

To bridge this diagnostic gap, several rapid microbiological techniques have emerged. Matrix-assisted laser desorption/ionization time-of-flight mass spectrometry (MALDI-TOF MS) enables pathogen identification directly from positive blood cultures within minutes, improving the accuracy and speed of microbiological workflows. Similarly, chromogenic media, semi-automated culture systems, and rapid biochemical identification panels shorten detection time and facilitate earlier optimization of therapy [42].

Biomarkers such as procalcitonin (PCT) and C-reactive protein (CRP) provide adjunctive information that may support early differentiation between bacterial and non-bacterial causes of inflammation, although they cannot replace microbiological testing. Emerging tools such as direct-from-sample molecular assays (e.g., rapid PCR panels for resistance genes) offer additional diagnostic value, particularly when resistant organisms are suspected [43,44].

Altogether, combining clinical assessment with Gram staining, microscopy, rapid culture-based methods, and early molecular testing enhances the ability of clinicians to identify pathogens more quickly and aids in selecting the most appropriate empiric therapy. This integrated diagnostic approach can improve patient outcomes and aligns with antimicrobial stewardship principles by enabling earlier de-escalation once definitive results are available [45,46].

## 5. Rapid Molecular Diagnostics for Antimicrobial Resistance

### 5.1. PCR-Based Techniques: GeneXpert System

Rapid determination of antimicrobial resistance is of paramount importance in critically ill patients, where delays in effective therapy are strongly associated with increased morbidity and mortality. Polymerase chain reaction (PCR)-based assays targeting resistance genes directly from pathological samples (e.g., tracheal aspirates, bronchoalveolar lavage in respiratory infections) offer clinicians timely results without requiring specialized laboratory expertise. These molecular approaches can complement traditional culture and susceptibility testing by significantly reducing diagnostic turnaround times.

In Western Romania, two rapid diagnostic platforms are currently available in clinical practice. Here we present one of them, while acknowledging that multiple technologies are in use worldwide.

GeneXpert^®^ System (Cepheid, USA)

The GeneXpert platform (developed by Cepheid, USA) is a rapid molecular diagnostic system based on real-time polymerase chain reaction (PCR) that utilizes automated cartridges for sample processing and pathogen detection. It is a critical tool for diagnosing infectious diseases, particularly tuberculosis (TB), as well as other infections such as mpox, SARS-CoV-2, and infections caused by antibiotic-resistant bacteria (e.g., carbapenemases). The GeneXpert Carba-R assay provides results in 45–60 min, detects major carbapenemase genes (KPC, NDM, OXA-48-like, VIM, IMP), and demonstrates sensitivity of 96–100% and specificity of 93–99% in Gram-negative isolates. These performance values are specific to the GeneXpert platform and to carbapenemase detection [45].

#### 5.1.1. Diagnostic Performance and Sensitivity/Specificity

For TB and Rifampicin Resistance (Xpert MTB/RIF): The GeneXpert MTB/RIF assay demonstrates a sensitivity of 90.3% for culture-confirmed TB cases and 76.9% for smear-negative cases, with a specificity of 99% [47]. A 2021 meta-analysis reported an overall sensitivity of 90.2% and specificity of 86.9% for both pulmonary TB (PTB) and extrapulmonary TB (EPTB), outperforming acid-fast bacilli (AFB) smear microscopy. For EPTB (e.g., TB lymphadenitis), sensitivity reaches 94% compared with fine-needle aspiration cytology (FNAC)/histopathology, enabling diagnosis in under 2 h [48].

For Other Infections: In COVID-19 diagnosis, the Xpert Xpress SARS-CoV-2 assay achieves 100% sensitivity and specificity compared with standard RT-PCR, making it ideal for low-throughput hospital settings with results in under 1 h. For mpox in the Democratic Republic of Congo (DRC), 240 GeneXpert machines have enhanced decentralized testing, supported by recent deliveries of 3500 cartridges for reliable diagnostics [49].

Comparison with Other Methods: GeneXpert outperforms conventional cultures (48–72 h turnaround) and is comparable to GenoType MTBDRplus for rifampicin resistance detection, while delivering results faster. The Xpert MTB/RIF Ultra version improves sensitivity for low-bacillary-load samples (e.g., EPTB), with 90% detection in pulmonary and extrapulmonary specimens [39].

#### 5.1.2. Implementation in Low and Middle-Income Countries

A 2021 systematic review found that GeneXpert increases TB case detection by 30–50% compared with smear microscopy, though its impact depends on local algorithms and logistics (e.g., sample transport). In Madagascar, combining GeneXpert with Loopamp MTBC increased case detection by 20%, but costs and maintenance remain barriers [50].

Recent Examples: In Kenya (Turkana, 2025), plans to deploy additional GeneXpert machines with X-ray aim to reduce TB mortality (9% in 2024) in areas with 3443 cases. In Pakistan, scaling to 774 GeneXpert units by 2026, supported by a USD 185M Global Fund investment, will enhance TB diagnosis. In Bhutan, Egypt, and Mongolia, integration into national systems has improved TB surveillance through connected databases [51].

#### 5.1.3. Clinical Impact in MDR Infections

Carbapenemase Detection (Xpert Carba-R): The Xpert Carba-R assay detects carbapenemase genes (KPC, NDM, VIM, OXA-48, IMP) in Gram-negative bacteria, with 96–100% sensitivity and 93–99% specificity [51,52,53]. A 2021 study validated its use in detecting Klebsiella pneumoniae carbapenemase variants, with results in 45 min, enabling rapid therapeutic decisions in ICU settings.

MRSA Detection: The Xpert MRSA/SA assay identifies *mec*A/*mec*C genes in respiratory samples, guiding the inclusion of MRSA-active agents (e.g., vancomycin, linezolid) or de-escalation when resistance genes are absent, reducing unnecessary antibiotic exposure.

Impact on Outcomes: In sepsis, GeneXpert’s rapid turnaround reduces time to effective therapy, lowering mortality by up to 20% in bloodstream infections when combined with stewardship. In Romania, where carbapenem-resistant Acinetobacter spp. prevalence is high (45% per your data), GeneXpert supports the timely use of novel agents like ceftazidime–avibactam.

Resistance-gene detection provides an important early signal for antibiotic selection; however, therapeutic decisions must integrate gene results with species identification, MIC confirmation, and local formulary constraints. For example, detection of blaKPC strongly predicts high-level carbapenem resistance and supports the early use of ceftazidime–avibactam or meropenem–vaborbactam, while avoiding carbapenem monotherapy. In contrast, for NDM-producing Enterobacterales, the first-line therapy is aztreonam–avibactam, the newly approved fixed-dose combination (EMBLAVEO^®^), which overcomes the hydrolytic activity of metallo-β-lactamases while inhibiting co-produced ESBL/*Amp*C enzymes. Cefiderocol remains a validated alternative.

The inoculum effect must be considered for ESBL and AmpC producers, where high bacterial burden can result in carbapenem-sparing regimens failing despite apparently favorable MICs. Likewise, gene–phenotype discordance can occur for OXA-48–like enzymes, which show highly variable carbapenem hydrolysis depending on species and expression level. For this reason, all molecular results should be validated with phenotypic susceptibility testing (MICs) once available.

Finally, panel menus are inherently limited, and off-panel organisms or resistance mechanisms (e.g., efflux pumps, porin loss) may remain undetected. Therefore, molecular findings should guide—not replace—clinical judgment and stewardship review (Table 2).

#### 5.1.4. Advantages and Limitations of Rapid PCR-Based Diagnostic Platforms (Table 3)

Clinical impact of rapid molecular diagnostics: Implementation of rapid molecular diagnostic panels has shown measurable clinical benefits in several ICU cohorts. In bloodstream infections, rapid identification and resistance-gene detection have been associated with a 17–36 h reduction in time-to-effective therapy [46,64]. Furthermore, syndromic testing has consistently demonstrated an increase in antimicrobial de-escalation rates by 22–45%, supporting antimicrobial stewardship goals and reducing unnecessary broad-spectrum antibiotic exposure [65,66]. These improvements underscore the practical value of integrating rapid diagnostics into ICU workflows.

**Table 3:** Advantages and Limitations.

Category	Description	Clinical Impact	Supporting Evidence
Speed	Results available in 45–60 min compared with 48–72 h for cultures	Early optimization of empiric therapy; faster escalation/de-escalation	Tsalik et al., 2018 [45] Zhang et al., 2024 [39]
Ease of Use	Minimal training required; fully automated	Expands access to rapid diagnostics outside tertiary centers	Centner et al., 2024 [49] Albert H et al., 2016 [67]
High Negative Predictive Value (NPV)	Reliable exclusion of major resistance genes when negative	Enables safe de-escalation and reduces broad-spectrum antibiotic use	Li et al., 2021 [51] Buchan et al., 2020 [65]
Detection of Key Resistance Genes	Identifies KPC, NDM, OXA-48-like, *mec*A/*mec*C	Earlier targeted therapy for MDR pathogens	Tsalik et al., 2018 [45] Li et al., 2021 [51]
Limited Scope	Does not detect all resistance mechanisms (e.g., efflux pumps, porin mutations)	May fail to predict phenotypic resistance	Tsalik et al., 2018 [45] Chakravorty et al., 2017 [50]
No Species Identification	Resistance genes may belong to various bacterial species	Requires culture confirmation for accurate therapy selection	Darie et al., 2022 [64] Buchan et al., 2020 [65]
Cost Constraints	Cartridge cost USD 20–65 per test, plus instrument maintenance	Financial barrier for widespread implementation in the ICU	WHO, 2024 [5] Albert H et al., 2016 [67]
False Positives/Negatives	FP: detection of non-viable DNA; FN: low bacterial load	Must be interpreted together with clinical findings and cultures	Huang et al., 2013 [68] Clark et al., 2013 [42]

### 5.2. UNYVERO^®^ System

The UNYVERO platform employs real-time multiplex polymerase chain reaction (PCR), enabling the simultaneous amplification of multiple nucleic acid targets within a single reaction. This technology allows for the detection of both microorganisms and resistance genes directly from clinical specimens. Within approximately 4–5 h, the system provides syndrome-specific pathogen and resistance gene results, as demonstrated for the severe pneumonia (HPN) panel, intra-abdominal infection (IAI) panel, implant and tissue infection (ITI) panel, and blood culture positive (BCU) panel cartridges This turnaround time applies specifically to the UNYVERO platform and should not be generalized to other rapid molecular systems. Dedicated cartridges are available for a wide range of infectious syndromes, including hospital-acquired pneumonia, intra-abdominal infections, urinary tract infections, skin and soft tissue infections, and bloodstream infections (positive blood cultures) [64].

Depending on the clinical presentation and the type of specimen collected, one of the pre-configured cartridges is selected (e.g., respiratory, urinary, intra-abdominal, skin/soft tissue, or positive blood cultures). The system thus follows a syndromic testing principle, identifying in a single assay one or more of the pathogens most frequently implicated globally in the respective infection type.

Advantages of the UNYVERO system include the following:

- Enhanced pathogen detection: Identifies clinically relevant pathogens that may be missed by conventional culture-based methods [65].
- Rapid results from challenging specimens: Delivers accurate results even in samples that are difficult to process by standard microbiology.
- Optimized antimicrobial therapy: By detecting both pathogens and resistance genes, it reduces the risk of ineffective antibiotic use [15].
- Improved clinical outcomes: Facilitates earlier targeted therapy, thereby reducing mortality and morbidity.
- Economic benefits: Early optimization of therapy and reduced length of hospital stay translate into substantial cost savings for healthcare systems.

Overall, the UNYVERO platform exemplifies the growing importance of syndromic molecular diagnostics in the management of severe infections, bridging the gap between empiric therapy and definitive culture-based results [69].

The simultaneous identification of pathogens and resistance genes enables clinicians to make empiric antibiotic therapy decisions in emergency settings with unprecedented speed and accuracy. By providing both taxonomic and resistance information within a short timeframe, molecular diagnostic platforms allow empiric therapy to be tailored more precisely to the patient’s infection profile.

In the presence of resistance genes, clinicians are guided to select only antibiotics demonstrated to be effective against the corresponding enzymatic resistance mechanisms, while avoiding classes to which the organism is predicted to be resistant. For example, the detection of carbapenemase genes rules out the use of carbapenems as monotherapy, given their lack of efficacy against carbapenemase-producing organisms. Instead, therapeutic choices must include either combination regimens or newer agents specifically designed to target such resistance mechanisms [70].

This approach not only reduces the likelihood of therapeutic failure but also limits unnecessary exposure to ineffective antibiotics, thereby supporting antimicrobial stewardship principles while improving clinical outcomes in critically ill patients.

### 5.3. Characteristics of Specific UNYVERO Panels

#### 5.3.1. Severe Pneumonia (HPN) Panel

The UNYVERO HPN panel is a multiplex PCR-based diagnostic tool specifically designed to accelerate pathogen identification and resistance gene detection in patients with suspected severe pneumonia. Its main characteristics are as follows:

High diagnostic accuracy: Sensitivity of 94% and specificity of 99.4%, enabling reliable detection of clinically significant pathogens [71].

Broad coverage: Detects more than 90% of the pathogens most frequently associated with severe pneumonia in critically ill patients [71,72].

Comprehensive pathogen and resistance marker detection: Identifies 21 pathogens (see Table 4) and 19 antimicrobial resistance markers (see Table 5).

Diverse specimen compatibility: Validated for use with sputum, tracheal or bronchial aspirates, and bronchoalveolar lavage fluid, thereby ensuring applicability across a wide range of clinical scenarios.

The combination of broad pathogen coverage and simultaneous resistance gene detection positions the UNYVERO HPN panel as an important diagnostic adjunct in the management of severe pneumonia, particularly in intensive care settings where timely and targeted therapy is crucial [70].

#### 5.3.2. Intra-Abdominal Infection (IAI) Panel

The UNYVERO IAI panel is a syndromic, multiplex PCR-based diagnostic tool developed to rapidly detect both pathogens and resistance markers directly from intra-abdominal samples. Its main characteristics are as follows:

High diagnostic performance: Demonstrates a sensitivity of 93.8% and a specificity of 99.7%, ensuring reliable detection of clinically relevant microorganisms [70].

Extensive coverage: Detects a broad spectrum of clinically relevant pathogens commonly implicated in intra-abdominal infections, which are associated with substantial morbidity and mortality in critically ill patients.

Comprehensive pathogen detection: Capable of identifying 92 bacterial species and 13 fungal pathogens, reflecting the polymicrobial nature of many intra-abdominal infections.

Resistance marker identification: Simultaneously detects 22 antimicrobial resistance markers, enabling early optimization of empiric therapy and supporting antimicrobial stewardship.

By combining broad-spectrum pathogen coverage with rapid resistance gene detection, the UNYVERO IAI panel provides clinicians with a powerful diagnostic adjunct that can significantly improve early therapeutic decisions and patient outcomes in intra-abdominal infections [58].

#### 5.3.3. Implant and Tissue Infection (ITI) Panel

The UNYVERO ITI panel is a multiplex PCR-based diagnostic assay designed to rapidly detect pathogens and resistance markers directly from clinical specimens obtained in cases of suspected tissue or implant-related infections. These infections often involve biofilm-forming microorganisms, making conventional culture-based diagnostics slow and sometimes unreliable.

Key characteristics of the ITI panel include the following:

High diagnostic accuracy: Overall sensitivity of 67%, with detection rates for key pathogens ranging between 75% and 100%, and a specificity of 97.8% [73].

Broad pathogen coverage Provides extensive detection of the primary microorganisms typically associated with soft-tissue and implant-related infections. Comprehensive target detection: Identifies 61 bacterial species, 10 fungal pathogens, and 19 antimicrobial resistance markers, reflecting the complexity and polymicrobial nature of implant-related infections.

Diverse specimen applicability: Validated for multiple sample types, including aspirated fluids, synovial fluid, tissue biopsies, swabs, catheter tips, transudates, drainage fluids, and bone fragments.

By providing simultaneous pathogen identification and resistance gene profiling, the ITI panel allows for rapid optimization of empiric therapy and earlier initiation of targeted treatment strategies. This has the potential to improve patient outcomes, particularly in orthopedic, prosthetic joint, and device-associated infections, where early and accurate diagnosis is critical [73].

#### 5.3.4. Blood Culture Positive (BCU) Panel

The UNYVERO BCU panel is a multiplex PCR-based syndromic diagnostic assay designed to rapidly identify pathogens and resistance markers directly from positive blood culture bottles. Bloodstream infections represent one of the most severe infectious disease emergencies, where rapid and targeted antimicrobial therapy is critical for patient survival.

Key characteristics of the BCU panel include the following:

High diagnostic accuracy: Sensitivity of 96.2% and specificity of 99.4% for the detection of bloodstream pathogens in positive blood culture bottles. However, these performance metrics must be interpreted in the context of specific assay limitations. First, the BCU system requires a minimum microbial load corresponding to the positivity threshold of automated blood culture systems, which may reduce sensitivity in low-level bacteremia. Second, off-panel organisms remain a limitation, as pathogens not included in the assay menu cannot be detected and require conventional culture for identification. These constraints underscore the need for careful clinical interpretation and complementary microbiological testing [73].

Rapid signal confirmation: Provides confirmation of blood culture positivity, enabling clinicians to act upon results earlier than with conventional culture-based identification.

Comprehensive pathogen detection: Capable of identifying 87 bacterial species and 28 fungal pathogens, covering the majority of clinically relevant organisms implicated in bacteremia and fungemia.

Resistance marker detection: Simultaneously detects 16 resistance genes, providing crucial information for early optimization of antimicrobial therapy.

Sample type: Validated for use directly with positive blood culture bottles, integrating seamlessly into routine microbiology workflows [71].

By combining broad pathogen coverage with rapid resistance profiling, the UNYVERO BCU panel offers a powerful diagnostic tool to accelerate clinical decision-making, reduce time to effective therapy, and improve outcomes in patients with sepsis and bloodstream infections [74].

## 6. Clinical Evidence and Local Data

Two major international studies have confirmed the accuracy and clinical utility of the UNYVERO platform in the rapid diagnosis of severe infections.

The first study, conducted at University College London, Royal Free Campus (UK), compared the performance of the UNYVERO HPN panel in patients with respiratory infections against conventional culture-based microbiology. The UNYVERO system demonstrated a specificity of 94.9% and a sensitivity of 88.8%, while identifying an average of 1.34 pathogens per sample, compared with only 0.47 pathogens per sample detected by traditional culture techniques. This discrepancy highlights the significant rate of false-negative results associated with culture methods. Moreover, the average turnaround time for conventional antimicrobial susceptibility testing was 53 h, underscoring the advantage of rapid molecular diagnostics in critically ill patients [43].

A second multicenter study, published in 2021 and performed across 11 academic centers in the United States, evaluated more than 2000 tracheal aspirates. The UNYVERO system achieved a specificity of 99.5% and a sensitivity of 91.4%, while also identifying pathogens that failed to grow in routine culture. These findings further support the role of syndromic molecular testing in improving diagnostic yield and accelerating targeted therapy [75].

Local epidemiological data were derived from a retrospective analysis conducted at the Department of Anaesthesia and Intensive Care of the Clinical Emergency Hospital “Pius Brînzeu” Timișoara, Romania. The dataset included all consecutive adult patients admitted between June 2022 and September 2024 from whom lower respiratory tract samples (tracheal aspirates or bronchoalveolar lavage) were collected based on clinical suspicion of nosocomial pneumonia. Samples were processed using the UNYVERO HPN panel according to manufacturer instructions. For polymicrobial detections, all pathogens reported by the panel were recorded without hierarchical weighting.

Carbapenem resistance in *Acinetobacter baumannii* isolates was determined using a combined approach: detection of carbapenemase genes (e.g., OXA-23-like, OXA-40-like) by the UNYVERO panel and confirmation of phenotypic resistance based on standard antimicrobial susceptibility testing performed by the hospital microbiology laboratory. Data were aggregated, anonymized, and analyzed descriptively using proportions and frequencies. Out of 229 processed samples, 66 (28.8%) were negative, whereas 163 (71.2%) yielded at least one pathogen included in the UNYVERO respiratory panel (Table 6). A notably high incidence of *Acinetobacter* spp. was observed, with 45% of isolates demonstrating carbapenem resistance.

Ethics Statement: This retrospective analysis used fully anonymized, aggregated laboratory data without patient identifiers. According to institutional policy, such datasets do not require individual informed consent. The study received approval/exemption from the Ethics Committee of the Clinical Emergency Hospital “Pius Brînzeu” Timișoara(CEHPBT) 569/08.10.2025, which confirmed that no additional ethical approval was required for the use of anonymized retrospective data.

It must be underscored that PCR-based diagnostic procedures are systematically implemented in the ICU of CEHPBT for critically ill patients with life-threatening infections, including sepsis and septic shock, particularly in those with hemodynamic instability, where rapid therapeutic decision-making is essential for improving survival outcomes. Following the availability of results, the attending physician tailors antimicrobial therapy by initiating novel broad-spectrum agents when resistance genes are detected, or, in their absence, by de-escalating to narrower-spectrum regimens.

As an original contribution, we constructed a comprehensive reference table (Table 6) synthesizing data from the international literature and the Summaries of Product Characteristics (SmPCs) of newly approved antimicrobials that have entered the pharmaceutical market in recent years. The antibiotics included are: ceftolozane–tazobactam (Zerbaxa^®^ Merck&Co., Inc., Rahway, NJ, USA), eravacycline (Xerava^®^, Tetraphase Pharmaceuticals, Watertown, MA, USA), ceftazidime–avibactam (Zavicefta^®^ Pfizer Europe MA EEIG, Brussels, Belgium), imipenem–relebactam (Recarbrio^®^ Merck Sharp & Dohme Corp, Rahway, NJ, USA), aztreonam–avibactam (Emblaveo^®^ Pfizer Europe MA EEIG, Brussels, Belgium), tedizolid (Sivextro^®^ Merck Sharp & Dohme Corp, Rahway, NJ, USA), dalbavancin (Xydalba^®^ Angelini Pharma S.p.A., Rome, Italy), and daptomycin (Cubicin^®^ Merck Sharp & Dohme Corp, Rahway, NJ, USA).

In parallel with the selection of antibacterial therapy, it remains critical to evaluate the risk of invasive fungal disease and to ensure the prompt initiation of appropriate antifungal treatment whenever indicated.

The therapeutic arsenal against MDR pathogens has recently expanded with several newly developed and EMA-approved antibiotics, which are available in Romania and the rest of the European Union. However, their use must be judicious, strictly guided by diagnostic results (PCR or culture-based). Indiscriminate or widespread empirical use without microbiological confirmation will inevitably lead to the rapid emergence of resistance, once again leaving clinicians with limited or no therapeutic options [76].

The susceptibility rates highlight the strengths and limitations of novel antibiotics against MDR pathogens, particularly carbapenem-resistant Enterobacteriaceae (CRE), *Pseudomonas aeruginosa* (CRPA), CRAB, *Stenotrophomonas maltophilia*, methicillin-resistant *Staphylococcus aureus* (MRSA), and *Clostridioides difficile*. For Gram-negative pathogens, agents such as cefiderocol, ceftazidime/avibactam, ceftolozane/tazobactam, imipenem/relebactam, and aztreonam/avibactam show robust activity (85–100% susceptibility) against CRE with KPC or OXA-48-like mechanisms, reflecting their design to counter β-lactamase-mediated resistance, notably, cefiderocol and aztreonam/avibactam demonstrate unique efficacy against metallo-β-lactamase (MBL)-producing CRE (85–95%), addressing a critical gap where other β-lactam/β-lactamase inhibitors fail (<10% susceptibility for MBLs). This makes them valuable in regions such as Romania, where MBL-producing strains are emerging. For CRPA, ceftolozane/tazobactam and imipenem/relebactam achieve high susceptibility (77–98.6% and 92–98%, respectively) in carbapenemase-negative isolates, but efficacy drops sharply against MBL-producing strains, underscoring the need for rapid diagnostics to identify resistance mechanisms. Cefiderocol maintains broader activity (80–98%) across CRPA, including MBL producers, positioning it as a versatile option for nosocomial pneumonia and bloodstream infections (BSIs). However, CRAB remains a challenge, with only eravacycline (80–95%) and cefiderocol (70–85%) showing reliable activity, reflecting the intrinsic resistance of A. baumannii to most β-lactams due to OXA-type carbapenemases and efflux pumps. S. maltophilia, inherently resistant to many agents, responds well to cefiderocol (85–95%) and moderately to eravacycline (70–85%), but other novel β-lactams are ineffective, necessitating alternative strategies such as trimethoprim-sulfamethoxazole or combination therapies. Gram-positive agents (vancomycin, linezolid, tedizolid, dalbavancin, daptomycin) exhibit near-universal activity (90–100%) against MRSA, with linezolid and tedizolid also effective against C. difficile (90–95%), supporting their role in skin/soft tissue infections (SSTIs) and hospital-acquired infections. However, their lack of Gram-negative activity limits their use in polymicrobial infections, which are common in intra-abdominal or implant-related infections, where combination with Gram-negative agents is often required.

The integration of rapid molecular diagnostics, such as the UNYVERO BCU panel, HPN panel, IAI panel, and ITI panels, is critical for interpreting Table 7′s susceptibility data in clinical practice. These platforms detect resistance genes (e.g., KPC, OXA-48, NDM) within 4–5 h, enabling clinicians to select antibiotics such as ceftazidime/avibactam for KPC-CRE or aztreonam/avibactam for MBL-CRE, avoiding ineffective agents such as ceftolozane/tazobactam in MBL-producing strains. For example, the high negative predictive value of UNYVERO BCU (99.8%) allows confident de-escalation to narrower-spectrum agents when resistance genes are absent, reducing selective pressure and supporting antimicrobial stewardship (AMS). Local data from Romania, showing 45% carbapenem resistance in A. baumannii, underscore the need for such diagnostics to guide eravacycline or cefiderocol use, as culture-based methods may take 48–72 h, delaying effective therapy in sepsis.

The susceptibility data reinforce the importance of AMS to preserve the efficacy of novel antibiotics. Indiscriminate use of broad-spectrum agents such as cefiderocol risks rapid resistance development, as seen with older carbapenems [16]. Table 7 supports a strategy of rapid diagnostics to initiate targeted therapy (e.g., ceftazidime/avibactam for KPC-CRE) followed by de-escalation to narrower agents (e.g., vancomycin for MRSA) when resistance profiles are clarified [11]. This approach reduces unnecessary antibiotic exposure, mitigates C. difficile risk, and aligns with WHO recommendations for combating AMR

Practice Points for ICU Teams

- Integrate rapid molecular diagnostics (e.g., HPN, BCU, Carba-R) into early sepsis evaluation to shorten time-to-effective therapy.
- Implement gene-guided antimicrobial selection, especially for KPC, NDM, and OXA-48-like results, with immediate consultation of AMS teams.
- Adopt structured de-escalation protocols that trigger antibiotic narrowing when rapid panel results and subsequent MIC data indicate susceptibility.
- Standardize empirical therapy choices based on local ICU antibiograms and syndrome-specific risk factors.
- Reinforce infection prevention bundles and ensure continuous auditing of device management and hand hygiene in high-risk ICU areas.

Research Agenda

- Evaluate the outcome impact of integrating rapid diagnostics with AMS algorithms across Romanian ICUs (mortality, LOS, antibiotic exposure).
- Compare diagnostic pathways (UNYVERO vs. GeneXpert vs. BioFire) in real-world ICU settings, focusing on concordance with culture/MIC results.
- Assess the cost-effectiveness of implementing rapid diagnostics in middle-income healthcare systems.
- Study gene–phenotype discordance, especially for OXA-48-like and AmpC, to refine therapeutic algorithms.
- Develop machine-learning models that combine rapid diagnostic results, local resistance patterns, and patient risk scores to guide individualized therapy.

## 7. Future Directions

Future research should focus on the following: Resistance Surveillance—expanding global and local surveillance (e.g., Romanian ICU data) to monitor emerging resistance (e.g., siderophore mutations in *cefiderocol*) and refine susceptibility breakpoints Combination Therapies—investigating optimal combinations (e.g., aztreonam/avibactam + meropenem-vaborbactam for MBL-CRE) to enhance efficacy and prevent resistance. Diagnostic Integration—validating newer UNYVERO panel iterations for emerging pathogens and resistance genes to further reduce diagnostic turnaround times. Clinical Trials—conducting real-world studies to confirm the efficacy of eravacycline and aztreonam/avibactam in complex infections, particularly bacteremia and polymicrobial settings.

## 8. Conclusions

The global rise in multidrug-resistant (MDR) infections continues to threaten the effectiveness of contemporary critical care, demanding more aggressive and coordinated action across diagnostic, preventive, and therapeutic domains. Evidence from international studies, supported by our local findings from Western Romania, demonstrates that the current pace of microbial evolution outstrips the capacity of traditional diagnostic and therapeutic approaches, underscoring the need for rapid, targeted, and integrated interventions.

Prevention strategies remain essential, with infection control bundles and strict compliance with hand hygiene, environmental cleaning, and device management showing consistent reductions in nosocomial infection rates. Antimicrobial stewardship (AMS) complements these measures by ensuring the rational use of antibiotics, preventing avoidable exposure, and slowing the emergence of resistant strains.

Rapid molecular diagnostics represent a pivotal advancement, providing actionable results within hours rather than days and enabling clinicians to initiate pathogen-directed therapy significantly earlier. Their integration into ICU workflows is especially critical in regions with high burdens of carbapenem-resistant organisms, as demonstrated by the substantial prevalence of resistant Acinetobacter spp. in our local cohort.

The expanding availability of novel antimicrobial agents offers new therapeutic possibilities; however, their clinical benefit depends on judicious use guided by reliable diagnostic information. Without stewardship and timely pathogen identification, resistance to these agents is likely to emerge rapidly.

Altogether, a consolidated strategy that aligns prevention bundles, stewardship principles, rapid molecular diagnostics, and responsible use of novel antimicrobials is essential to improve patient outcomes and protect the effectiveness of existing therapies. Strengthening these pillars—through sustained investment, training, and system-level implementation—remains critical to mitigating the impact of MDR infections in intensive care units and safeguarding future global antimicrobial efficacy.

## Figures and Tables

**Table 2:** Resistance genes and recommended antimicrobial pathways.

Resistance Gene Detected	Likely Mechanism/Implication	First-Line Therapy	Alternative Therapy	Key Caveats	Supporting Evidence
KPC	KPC carbapenemase → high-level carbapenem resistance	Ceftazidime–avibactam	Meropenem–vaborbactam; Imipenem–relebactam	MIC confirmation required; avoid carbapenem monotherapy; inoculum effect possible	Van Duin et al. [54]; Shields RK et al. [55];
NDM	Metalo-β- lactamase → carbapenem + ceftazidime–avibactam resistance	Aztreonam + avibactam	Cefiderocol	Gene/phenotype discordance may occur; confirm susceptibility	Sangiorgio et al. [56]; Falagas et al. [57];
OXA-48-like	OXA-48 carbapenemase; variable carbapenem hydrolysis	Ceftazidime–avibactam	High-dose meropenem; cefiderocol	Species- dependent expression; confirm phenotype	Tamma et al. [58]; Wang et al. [59];
*mec*A/*mec*C	MRSA; β-lactam resistance	Vancomycin; Daptomycin	Linezolid; Ceftaroline	Avoid β-lactams except ceftaroline;	Liu et al. [60];
ESBL genes (*CTX*-M)	ESBL-producing Enterobacterales	Carbapenem (merpenem)	Piperacillin–tazobactam; ceftolozane–tazobactam	Inoculum effect; avoid PTZ in severe sepsis unless MIC ≤ 8	Tamma et al. [58]; Rodrigues-Bano et al. [61];
*Amp*C genes	AmpC hyperproduction → cephalosporin resistance	Carbapenem	Cefepime (if MIC ≤ 2 mg/L)	Risk of treatment failure with cefepime if high inoculum	Tekele et al. [62]; Rezzoug et al. [63];
Cabapenemase gene negative (wild-type profile)	No major resistance gene detected	De-escalation to narrower β-lactams	Cephalosporins; β-lactam/β-lactamase inhibitors	Must confirm susceptibility; PCR does not detect efflux/porin loss	Tamma et al. [58];

**Table 4:** Pathogens identified by the UNYVERO respiratory panel.

Gram-Positive Bacteria	Enterobacteriaceae	Non-Fermentative Bacteria	Other Bacteria/Fungi
*Staphylococcus aureus* *Streptococcus pneumoniae*	*Citrobacter freundii* *Escherichia coli* *Enterobacter cloacae* complex *Klebsiella aerogenes (Enterobacter aerogenes)* *Proteus* spp. *Klebsiella pneumonia* *Klebsiella oxytoca* *Klebsiella variicola* *Serratia marcescens* *Morganella morganii*	*Moraxella catarrhalis* *Pseudomonas aeruginosa* *Acinetobacter baumannii* complex *Stenotrophomonas maltophilia* *Legionella pneumophila*	*Pneumocystis jirovecii* *Haemophilus influenzae* *Mycoplasma pneumoniae* *Chlamydia* (*Chlamydophila*) *pneumoniae*

**Table 5:** Antimicrobial resistance genes detected by the UNYVERO Respiratory Panel.

Antibiotic Resistance	Detected Gene
Macrolides/Lincosamides	*erm*B
Oxacillin	*mec*A, *mec*C
Penicillins	*tem*, *shv*
Third-generation cephalosporins	*ctx*-M
Carbapenems	kpc, imp, ndm, oxa-23, oxa-24/40, oxa-48, oxa-58, vim
Sulfonamides	sul1
Fluoroquinolones	*gyr*A83, *gyr*A87

**Table 6:** Pathogens identified using the UNYVERO respiratory panel in the ICU of CEHPBT, 2022–2024.

Identified Pathogens	Percentage (%)	Number of Isolates
*Acinetobacter baumannii* complex	20.4	72
*Pseudomonas aeruginosa*	18.41	65
*Klebsiella pneumoniae*	16.72	59
*Staphylococcus aureus*	7.93	28
*Stenotrophomonas maltophilia*	7.65	27
*Proteus* spp.	7.37	26
*Escherichia coli*	6.23	22
*Enterobacter cloacae* complex	3.4	12
*Streptococcus pneumoniae*	2.55	9
*Haemophilus influenzae*	2.55	9
*Klebsiella oxytoca*	1.98	7
*Serratia marcescens*	1.7	6
*Moraxella catarrhalis*	1.42	5
*Citrobacter freundii*	0.85	3
*Morganella morgani*	0.28	1
*Legionella pneumophila*	0.28	1
*Pneumocystis jirovecii*	0.28	1
*Klebsiella aerogenes* (*E. aerogenes*)	0	0
*Klebsiella variicola*	0	0
*Mycoplasma pneumoniae*	0	0
*Chlamydia* (*Chlamydophila*) *pneumoniae*	0	0

**Table 7:** Susceptibility of MDR pathogens to new antibiotics (adapted from multidrug-resistant Gram-negative bacterial infections) [77].

	*CR Enterobacteriacee*				*Pseudomonas a* *eruginosa*		CR *A. baumannii* CRAB	*S. maltophilia*	*MRSA*	*C. Diff.*
	OXA-48-like+	KPC+	MBL+	CR Cabapenemase —negative	MBL+	CR Cabapenemase —negative				
Cefiderocol										
Ceftazidime/ Avibactam										
Ceftolozane/ Tazobactam										
Imipenem/ Relebactam										
Eravacyline										
Aztreonam/ Avibactam										
Vancomycin										
Linezolid										
Tedizolid										
Dalbavancin										
Daptomycin										

Legend: Green = over 90% of strains are susceptible to this antibiotic; Yellow = between 50% and 90% of strains are susceptible; Red = 0–50% of strains are susceptible. CR: carbapenem-resistant; MRSA: methicillin-resistant Staphylococcus aureus; C. diff.: Clostridioides difficile.

## Data Availability

The data are encapsulated within this article. Further details can be obtained upon request from either the primary author or the corresponding author.
